# ε2 allele and ε2-involved genotypes (ε2/ε2, ε2/ε3, and ε2/ε4) may confer the association of *APOE* genetic polymorphism with risks of nephropathy in type 2 diabetes: a meta-analysis

**DOI:** 10.1186/s12944-020-01307-6

**Published:** 2020-06-13

**Authors:** Jikang Shi, Zhaorui Cheng, Shuang Qiu, Heran Cui, Yulu Gu, Qian Zhao, Yaxuan Ren, He Zhang, Helin Sun, Yunkai Liu, Yong Li, Yichun Qiao, Yueyang Hu, Yawen Liu, Yi Cheng

**Affiliations:** 1 Department of Epidemiology and Biostatistics, School of Public Health, Jilin University, Changchun, 130021 Jilin China; 2 Class of Clinical Medicine of English Teaching, Grade 2016, Ottawa-Shanghai Joint School of Medicine, Shanghai Jiao Tong University, Shanghai, 200023 China; 3 Institute of Translational Medicine, The First Hospital of Jilin University, Changchun, 130021 Jilin China; 4 Department of children & adolescence health, School of Public Health, Jilin University, Changchun, 130021 Jilin China

**Keywords:** Diabetic nephropathy, Type 2 diabetes, Apolipoprotein E, Polymorphism, Risk, Association

## Abstract

**Background:**

Diabetic nephropathy (DN) contributes to end-stage renal failure. Microvascular injury resulted from reactive oxygen species is implicated in the pathogenesis of DN. Genetic polymorphism of Apolipoprotein E (APOE) influences the antioxidative properties of the protein. The relationship of *APOE* polymorphism with the risks of nephropathy in type 2 diabetes (T2DN) remains elusive.

**Methods:**

An up-to-date meta-analysis was conducted on the basis of studies selected from PubMed, WanFang database, Embase, Vip database, Web of Science, Scopus, and CNKI database.

**Results:**

A total of 33 studies conferring 3266 cases and 3259 controls were selected on the basis of criteria of inclusion and exclusion in this meta-analysis. For *APOE* alleles, the pooled odds ratio (OR) of ε2 vs. ε3 was 1.89 (95% confidence intervals [95% CI]: 1.49–2.38, *P* < 0.0001). With regard to *APOE* genotypes, ε2/ε2, ε2/ε3, and ε2/ε4 increased the risk of T2DN (ε2/ε2 vs. ε3/ε3: OR = 2.32, 95% CI: 1.52–3.56, *P* = 0.0001; ε2/ε3 vs. ε3/ε3: OR = 1.97, 95% CI: 1.50–2.59, *P*<0.0001; ε2/ε4 vs. ε3/ε3: OR = 1.69, 95% CI: 1.18–2.44, *P* = 0.0046).

**Conclusions:**

This meta-analysis found that the *APOE* ε2 allele and the ε2-involved genotypes (ε2/ε2, ε2/ε3, and ε2/ε4) are the risk factors of T2DN.

## Background

Diabetic nephropathy (DN) contributes to end-stage renal failure [1]. Microvascular injury resulted from reactive oxygen species is implicated in the pathogenesis of DN [2, 3]. Elucidating risk factors of DN, such as genetic and environmental factors, is needed for controlling this disease.

Genetic factors complicated in DN etiology confer useful insights into the etiology of the disease [4]. Oxidative stress is also involved in the complex web of pathological events that confer susceptibility to DN [5, 6]. Excessive generation of reactive oxygen species (ROS) gives rise to imbalanced redox signaling, resulting in renal injury on the long term; moreover, oxidative stress is also linked to changes in the structure and function of apolipoprotein E (APOE), as its coding gene is implicated in DN pathology [7, 8]. Two single nucleotide polymorphisms (SNPs) (rs7412 and rs429358) existing on exon 4 of *APOE* gene combine to generate three major alleles: ε3 is characterized by cytosines in both positions, while substitution rs7412C > T defines ε2 and rs429358C > T determines ε4. The two SNPs confer APOE3 with arginine at residue 158 and cysteine on residue 112, APOE2 carrying cysteine on both positions, and APOE4 carrying arginine on both positions. Moreover, combinations of these alleles generate six *APOE* haplotypes (ε2/ε2, ε2/ε3, ε2/ε4, ε3/ε3, ε3/ε4, and ε4/ε4). Allele variation in *ApoE* locus accounts for 0–20% of ε2, 60–90% of ε3, and 10–20% of ε4, respectively [9]. Allele ε3 is accepted as “wild-type” as it is the most common, and ε2 and ε4 are variants. The association between the two SNPs and T2DN risk is conflicting. Lin et al. found that ε2 polymorphism increased the susceptibility to T2DN in Asian population [10]. ε2 carriers and ε3/ε4 genotype carriers had increasing risks of developing T2DN [11]. However, the differences in sample sizes, sample sources, disease status, genotyping method, and other uncontrolled factors generate the above disagreeing results.

Meta-analysis, featured in summarizing results quantitatively from a wide range of studies, is a powerful method of statistical analysis, increasing the sample size to reduce false-negative and false-positive associations caused by random errors. Notably, new studies on associations between *APOE* polymorphism and T2DN risks have been issued since Li et al. published their meta-analysis [12]. Therefore, an up-to-date meta-analysis was performed to further investigate the association by including these new published articles.

## Methods

### Articles search

The meta-analysis was conducted by searching the relative articles published before July 31, 2019 from PubMed, WanFang database, Embase, Vip database, Web of Science, Scopus, and CNKI database. The combinations of keywords were used for searching PubMed, Embase, Web of Science, Scopus were ([“APOE” OR “Apolipoprotein E”] AND [“Diabetic nephropathy”]). Furthermore, the equivalent Chinese keywords were utilized for searching the Chinese databases.

### Inclusion/exclusion criteria

The articles selected in the meta-analysis were based on inclusion criteria (case–control design; type 2 DM with DN; and association of *APOE* with DN risks) and the exclusion criteria (case reports or reviews; duplicate reports; type 1 DM; and missing data of allele or genotype frequencies).

### Data extraction and quality assessment

The information from the included articles was extracted, such as the last name of first author and data of *APOE* allele or genotype.

According to the Newcastle-Ottawa scale (NOS), the quality of the included articles was evaluated. If an included article met a condition, a score of one point was allocated; otherwise, no point (0 score) was allocated. Each of the included articles was awarded the sum of all points (total Quality Score) [13]. Moreover, the quality of these articles was evaluated by the two investigators (Zhaorui Cheng and Jikang Shi) independently. If an agreement for an included article was not reached by the two investigators, the third investigator (Shuang Qiu) settled inconformity finally. Low-quality articles were also selected to avoid selection bias.

### Statistical analysis

Chi-square test of goodness of fit was used for evaluating Hardy–Weinberg equilibrium (HWE) for each included article among control groups, and HWE was rejected when *P* < 0.05. The strength of association between *APOE* polymorphisms and T2DN risks was assessed using Odds ratios (OR) and 95% confidence intervals (95% CI) owing to binary outcome variable. Both Chi-square test-based Q-statistic and quantified by *I*^2^-statistic were adopted to evaluate heterogeneity. Because genotype can represent the combined effect of alleles, the comparisons of *APOE* genotypes were performed. For heterogeneity between studies given by I squared > 50%, random-effect models were applied; otherwise, if I squared < 50%, fixed-effect models were used [14]. Subgroup analyses were conducted to find main heterogeneity sources. Meta-regression was carried out to further reveal heterogeneity sources and the contribution to heterogeneity. Sensitivity analysis was conducted to evaluate the stability of overall results. Publication bias was examined by funnel plots, and quantified using the Begg’s and Egger’s tests: *P* < 0.05 was considered significant publication bias [15]. Bonferroni correction was carried out in multiple comparison; thus, *P* < 0.025 was considered as statistically significant. R Studio (Version 1.1.383) (RStudio, Inc., MA, USA) for Windows was used for all data management and analyses.

### Trial sequential analysis (TSA)

Dispersed data and repeated significance testing give rise to an increased risk of random error in traditional meta-analysis. TSA adjusts threshold for statistical significance, reducing the risk of type I error by required information size (RIS). In addition, TSA is used to estimate statistical reliability. In the meta-analysis, TSA software (TSA, version 0.9.5.5; Copenhagen Trial Unit, Copenhagen, Denmark, 2016) was used. The overall type I error was set at 5%, the statistical power was 80%, and the relative risk was reduced by 20% [16]. When the Z-curve crossed trial sequential monitoring boundary or RIS was reached, additional studies were not required; otherwise, additional studies were required.

## Results

### Characteristics of included articles

**Fig. 1:**
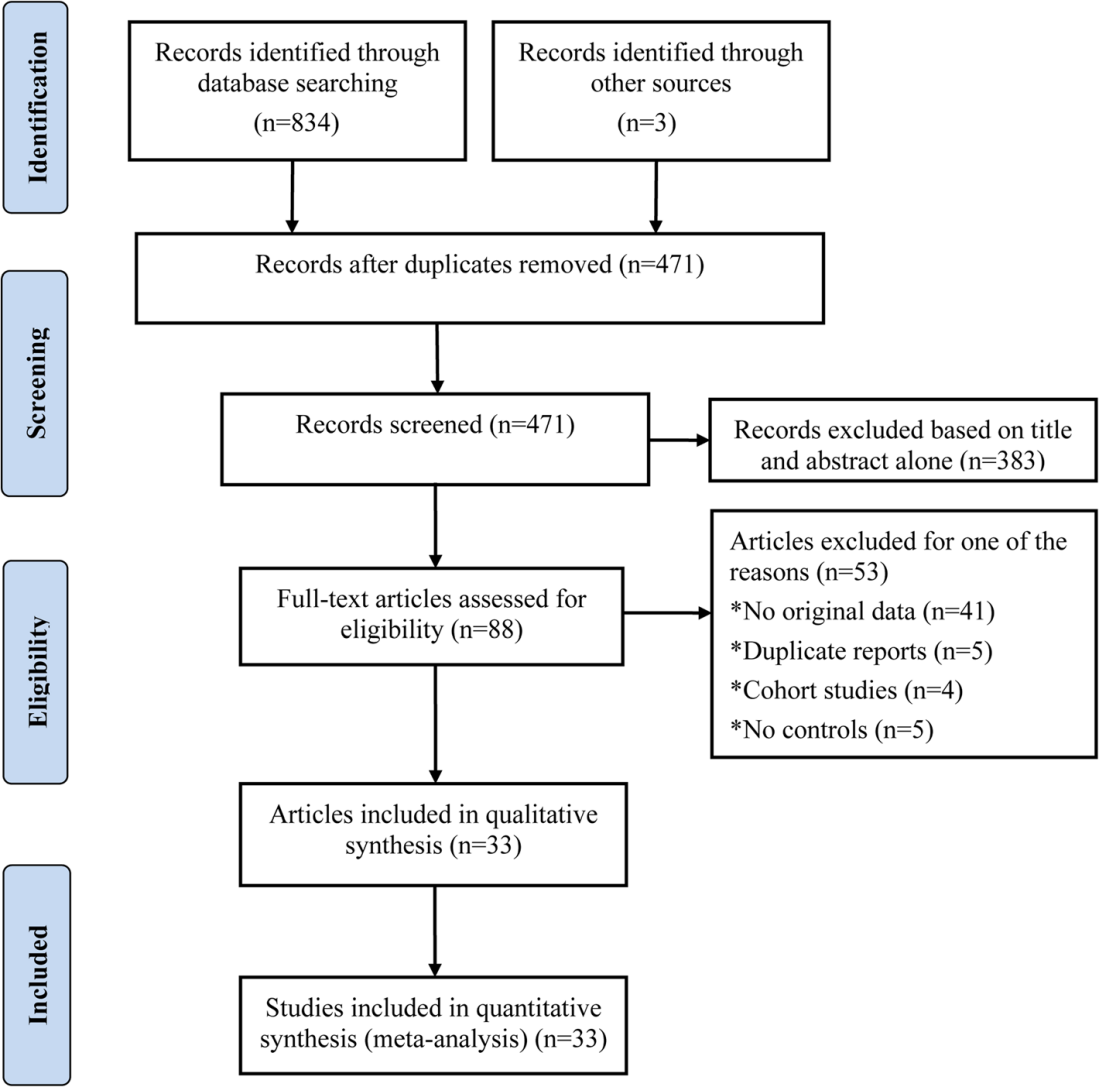
Flow chart of literature identification and selection

A total of 33 eligible articles were eventually chosen, after abstracts and full texts of 837 published articles originally collected were scrutinized according to the inclusion and exclusion criteria [17–49], thereby conferring 3266 cases and 3259 controls in this meta-analysis (Fig. 1) (Table 1).

**Table 1:** Main characteristics of the included studies

Study	Year	Region	Ethnicity	Genotyping method	Sample size (case/control)		Quality score	HWE Y/N	*ApoE* ε2 (n)		*ApoE* ε3 (n)		*ApoE* ε4 (n)		
									case	control	case	control	case	control	
Horita et al. [17]	1994	Japan	Asian	Flat gel isoelectric focusing	57/	398	7	Y	11	25	87	669	16	102	
Eto et al. [18]	1995	Japan	Asian	Flat gel isoelectric focusing	146/	135	5	Y	21	7	235	229	36	34	
Kimura et al. [19]	1998	Japan	Asian	PCR	81/	96	7	Y	7	10	143	154	12	28	
Zhang et al. [20]	1999	China	Asian	PCR	57/	40	6	Y	34	11	62	53	18	16	
Xiang et al. [21]	1999	China	Asian	PCR	46/	84	8	Y	12	9	71	137	9	22	
Ha et al. [22]	1999	Korea	Asian	PCR	74/	93	7	Y	18	8	119	163	11	15	
Akarsu et al. [23]	2000	Turkey	Turkish	PCR	24/	22	7	Y	11	3	33	35	4	6	
Dai et al. [24]	2000	China	Asian	PCR	88/	32	5	Y	14	5	143	54	19	5	
Shen et al. [25]	2002	China	Asian	PCR	159/	106	5	Y	38	11	250	186	30	15	
Zhang et al. [26]	2002	China	Asian	PCR	58/	56	7	Y	17	4	86	94	13	14	
Liu et al. [27]	2003	China	Asian	PCR	218/	80	7	Y	40	12	351	135	45	13	
Park et al. [28]	2004	Korea	Asian	PCR	48/	70	6	Y	12	3	79	123	5	14	
Liu et al. [29]	2004	China	Asian	PCR	56/	28	5	Y	15	2	87	49	10	5	
Xiong et al. [30]	2005	China	Asian	PCR	33/	32	6	Y	7	8	51	51	8	5	
Hua et al. [31]	2006	China	Asian	FRET-RELP	52/	50	7	N	23	12	160	160	17	28	
Guo et al. [32]	2006	China	Asian	PCR	32/	25	5	N	18	4	42	42	4	4	
Ng et al. [33]	2006	China	Asian	PCR	366/	386	8	Y	83	66	594	656	55	50	
Zhang et al. [34]	2007	China	Asian	PCR	40/	38	6	Y	9	2	61	69	10	5	
Pan et al. [35]	2007	China	Asian	PCR	113/	97	7	Y	17	20	172	163	37	11	
Ilhan et al. [36]	2007	Turkey	Turkish	PCR	37/	71	7	N	3	14	63	118	8	10	
Kwon et al. [37]	2007	Korea	Asian	PCR	36/	58	5	Y	7	9	61	92	4	15	
Leiva et al. [38]	2007	Chile	Latin	PCR	56/	29	7	Y	1	1	102	42	9	15	
Rouzi et al. [39]	2008	China	Asian	PCR	36/	17	6	N	16	4	52	26	4	4	
Erdogan et al. [40]	2009	Turkey	Turkish	PCR	46/	56	7	Y	5	4	80	96	7	12	
Xiang et al. [41]	2010	China	Asian	PCR	177/	41	5	Y	57	6	279	68	18	8	
Reis et al. [42]	2011	Turkey	Turkish	PCR	106/	110	7	Y	7	25	194	176	11	19	
Sun et al. [43]	2013	China	Asian	PCR	228/	243	7	Y	54	48	357	417	45	21	
Satirapoj et al. [44]	2013	Thailand	SE Asian	PCR	115/	115	6	Y	24	17	196	188	10	25	
Wang et al. [45]	2014	China	Asian	PCR	63/	57	8	Y	28	6	79	83	19	25	
Luo et al. [46]	2016	China	Asian	PCR	45/	35	5	Y	18	4	36	61	36	5	
Atta et al. [47]	2016	Egypt	Arabian	PCR	45/	45	7	N	45	24	27	45	18	21	
Jiang et al. [48]	2017	China	Asian	Genotyping chip	429/	416	8	N	74	33	708	699	76	100	
Karimoei et al. [49]	2017	Iran	Persian	PCR	99/	98	8	Y	14	10	163	146	21	40	

### Association of the *APOE* alleles with T2DN risks

**Fig. 2:**
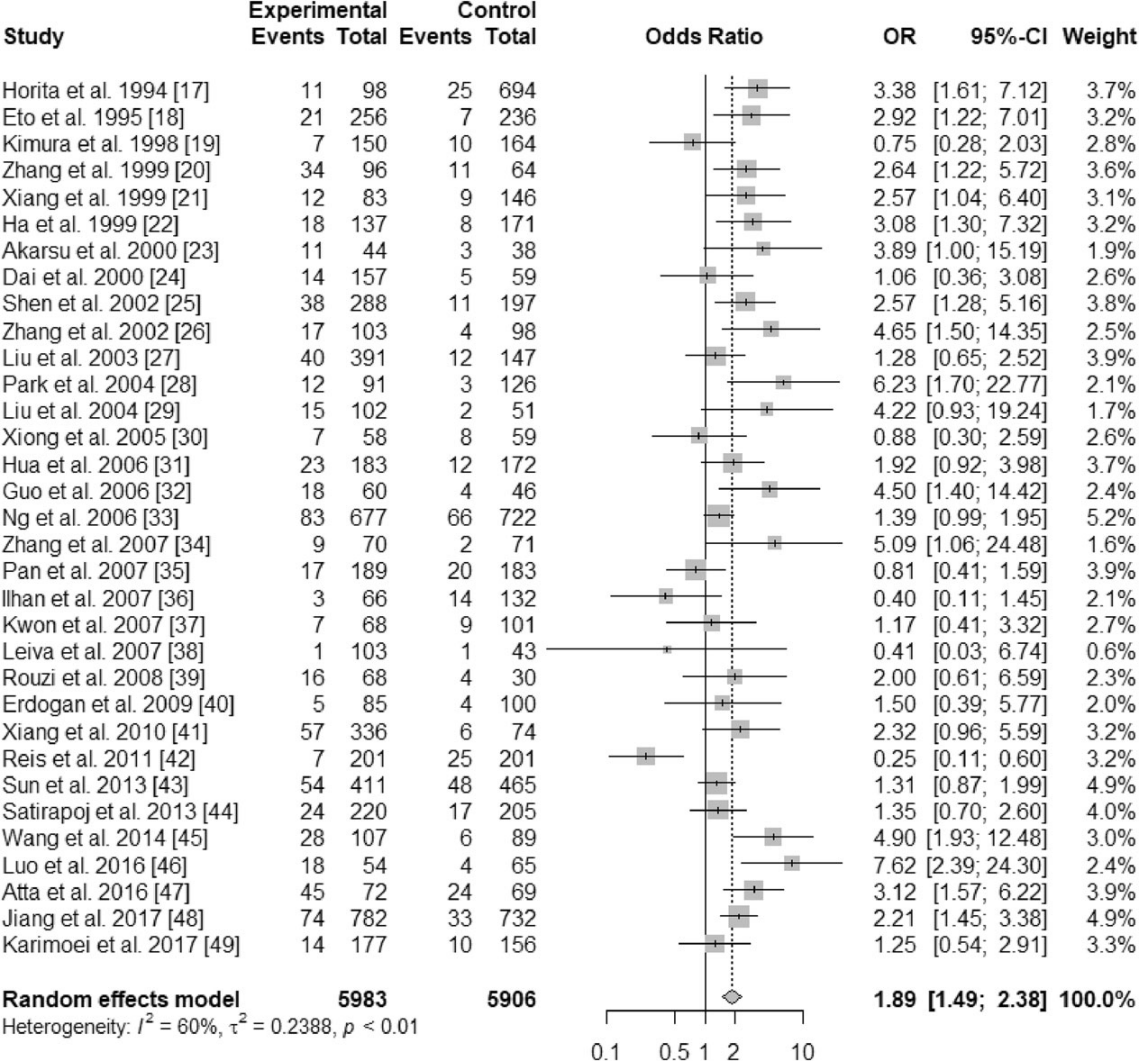
Forest plot for association between nephropathy in type 2 diabetes risk and *ApoE ε*2 allele vs. *ε*3 allele based on a random-effects model

**Fig. 3:**
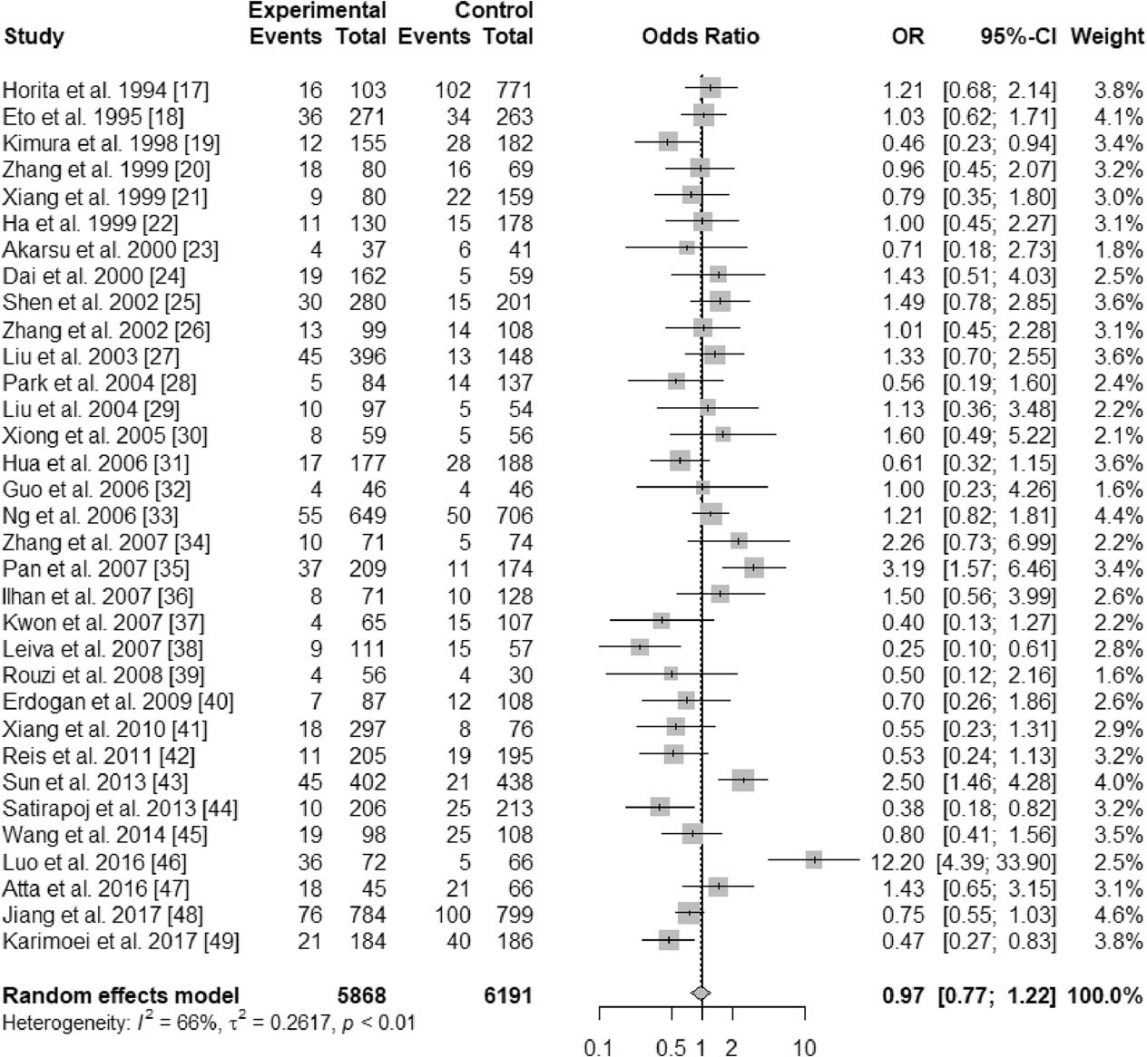
Forest plot for association between nephropathy in type 2 diabetes risk and *ApoE ε*4 allele vs. *ε*3 allele based on a random-effects model

A significant heterogeneity was found in ε2 vs. ε3 allele (*I*^2^ = 60%, *P* < 0.01) and in ε4 vs. ε3 allele (*I*^2^ = 66%, *P* < 0.01). Random-effects model was used in ε2 vs. ε3 (pooled OR = 1.89; 95% CI: 1.49–2.38; *P*<0.0001) (Fig. 2) and in ε4 vs. ε3 (pooled OR = 0.97; 95% CI: 0.77–1.22; *P* = 0.7948) (Fig. 3). Thus, ε2 allele is regarded as a risk factor of T2DN, and ε4 is not a protective factor.

### Association between *APOE* genotypes and T2DN risks

**Fig. 4:**
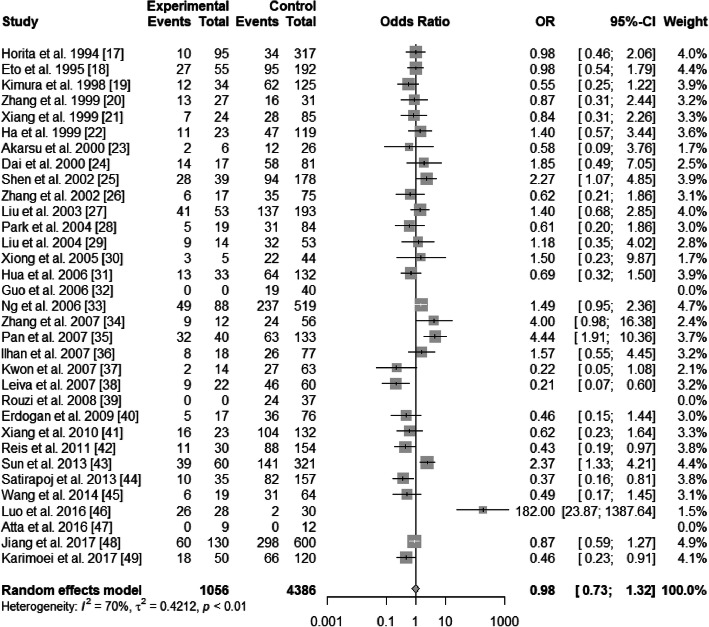
Forest plot for association between nephropathy in type 2 diabetes risk and *ApoE* genotype *ε*3/*ε*4 vs. *ε*3/*ε*3 genotype based on a random-effects model

**Fig. 5:**
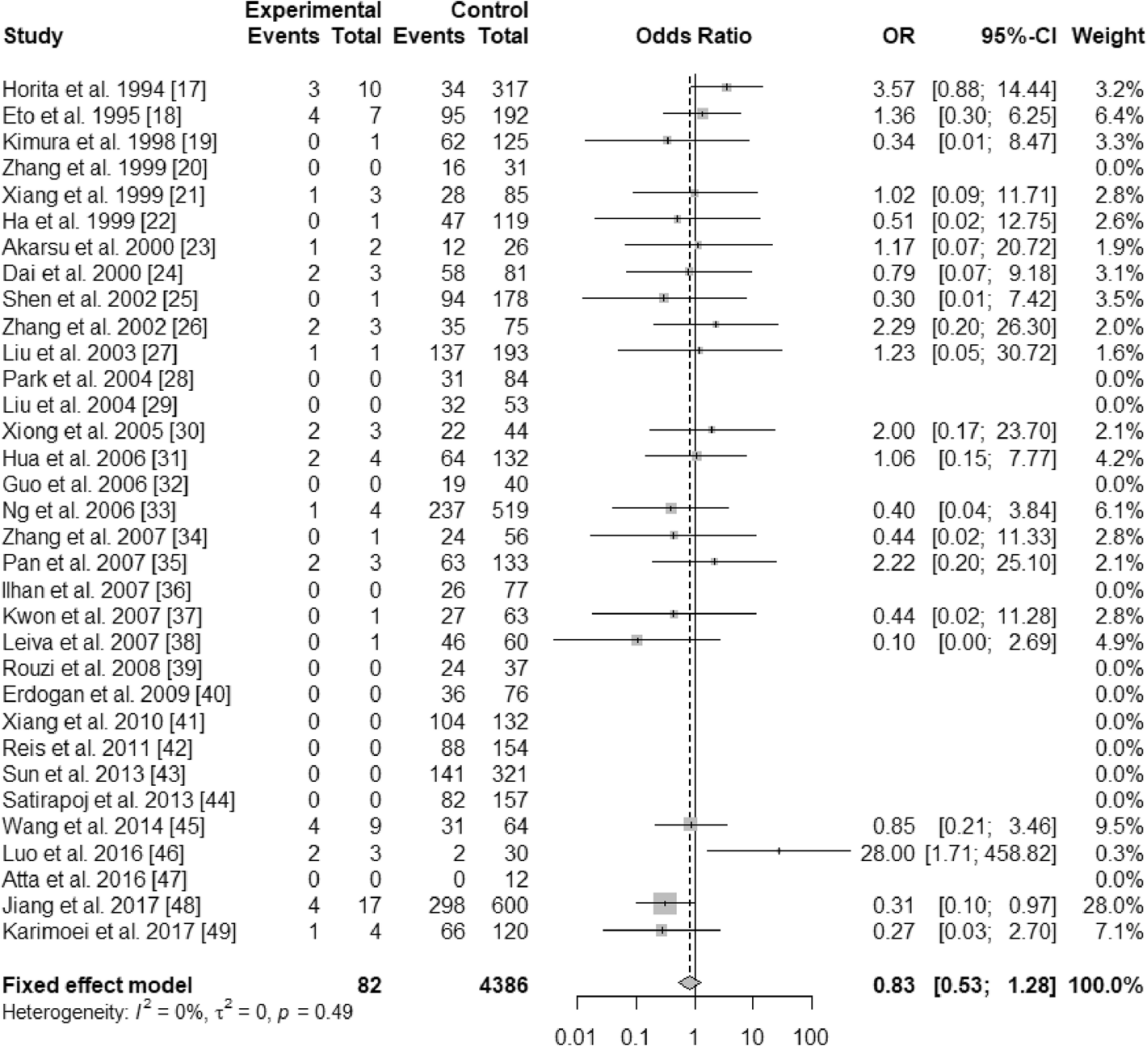
Forest plot for association between nephropathy in type 2 diabetes risk and *ApoE* genotype *ε*4/*ε*4 vs. *ε*3/*ε*3 genotype based on a fixed-effects model

**Fig. 6:**
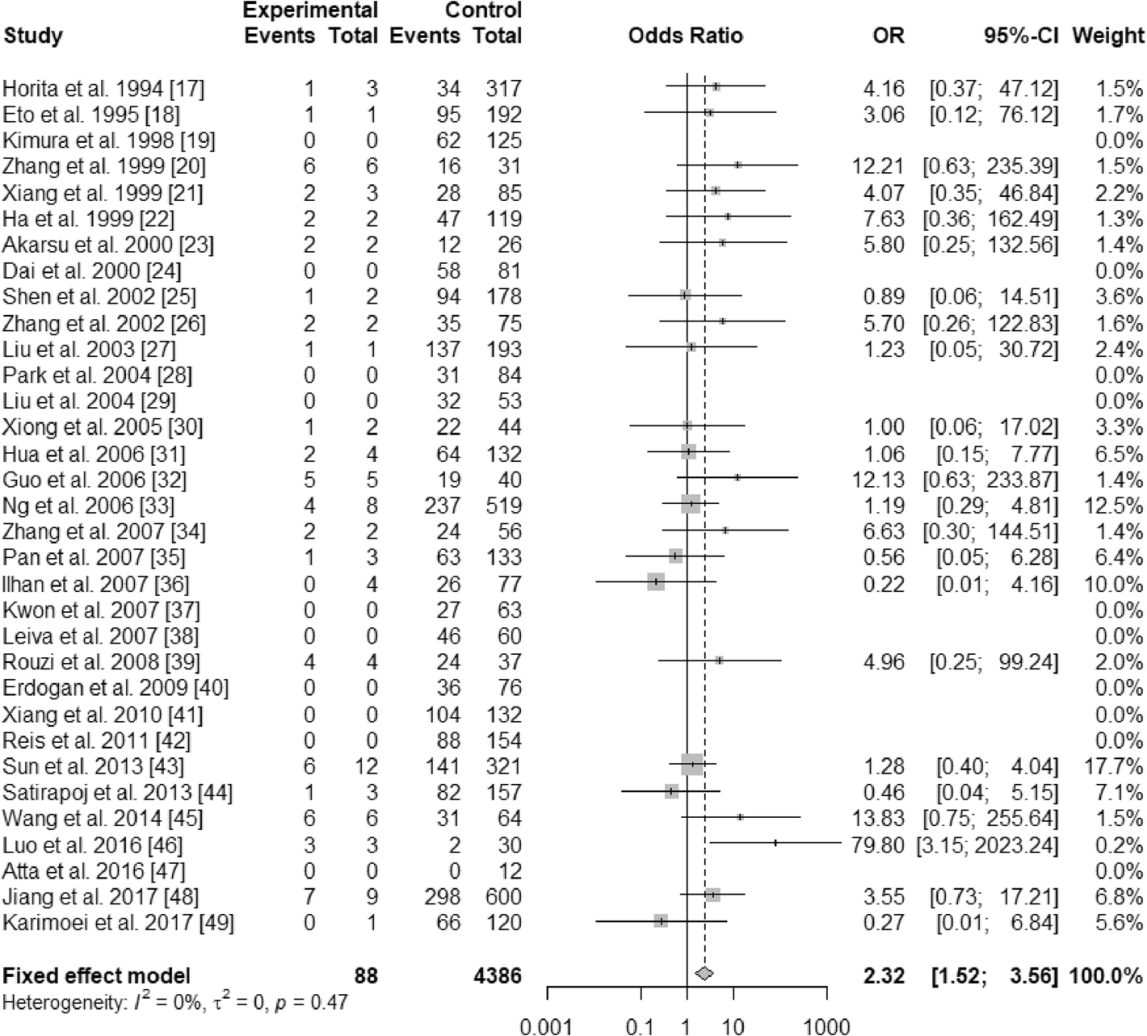
Forest plot for association between nephropathy in type 2 diabetes risk and *ApoE* genotype *ε*2/*ε*2 vs. *ε*3/*ε*3 genotype based on a random-effects model

**Fig. 7:**
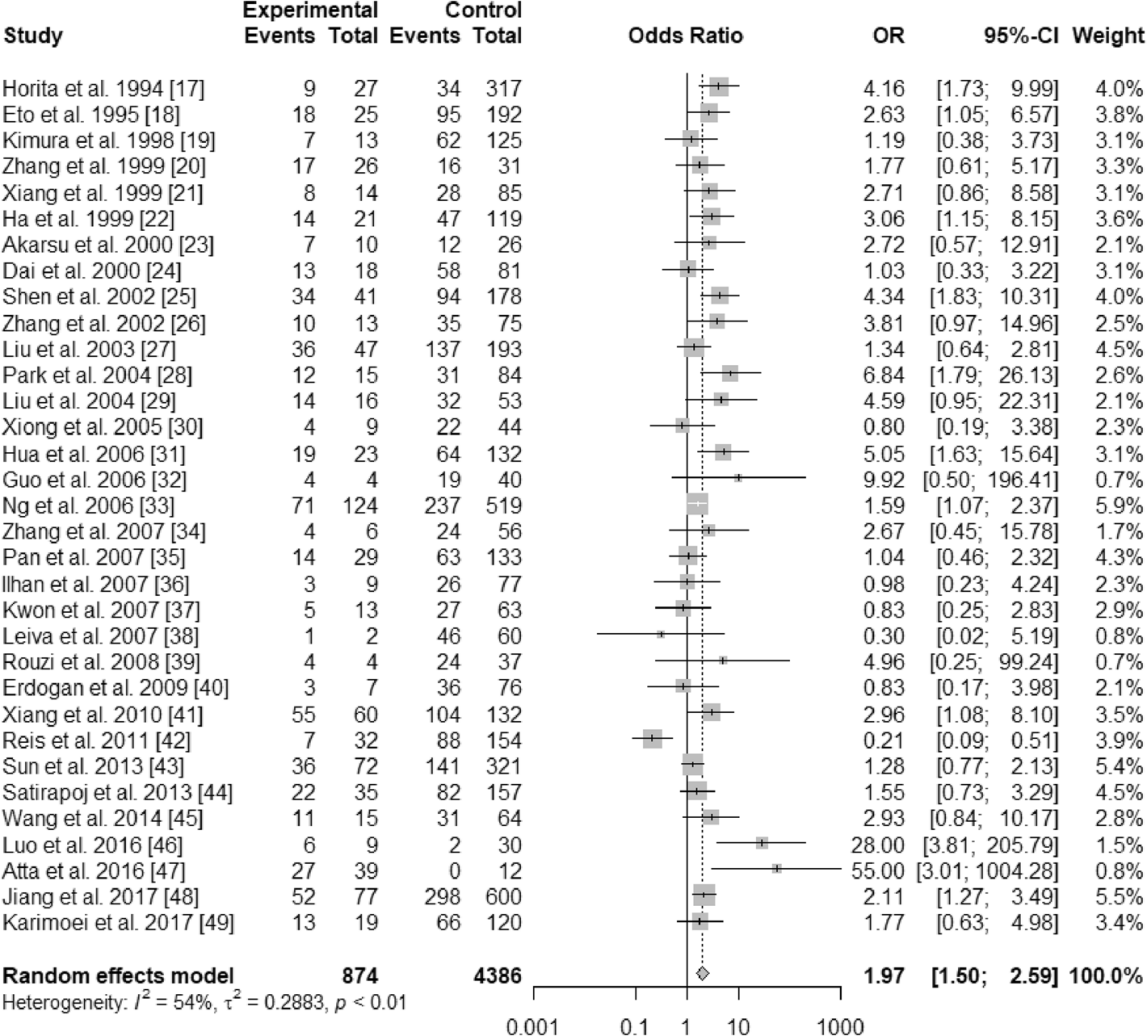
Forest plot for association between nephropathy in type 2 diabetes risk and *ApoE* genotype *ε*2/*ε*3 vs. *ε*3/*ε*3 genotype based on a fixed-effects model

**Fig. 8:**
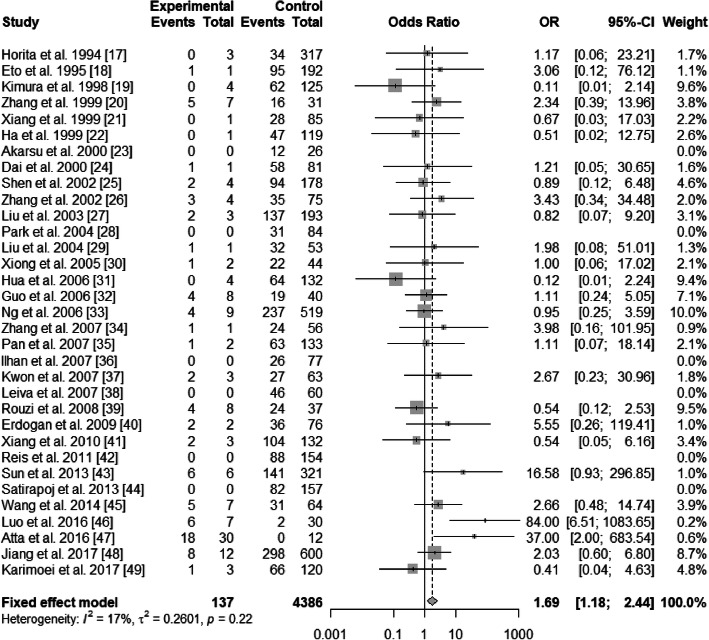
Forest plot for association between nephropathy in type 2 diabetes risk and *ApoE* genotype *ε*2/*ε*4 vs. *ε*3/*ε*3 genotype based on a fixed-effects model

There existed significant heterogeneity in ε2/ε3 and ε3/ε4 (ε2/ε3 vs. ε3/ε3: *I*^2^ = 54%, *P* < 0.01; ε3/ε4 vs. ε3/ε3: *I*^2^ = 70%, *P*<0.01), but not existed heterogeneity in ε2/ε2, ε2/ε4, and ε4/ε4 (ε2/ε2 vs. ε3/ε3: *I*^2^ = 0%, *P* = 0.47; ε2/ε4 vs. ε3/ε3: *I*^2^ = 17%, *P* = 0.22; ε4/ε4 vs. ε3/ε3: *I*^2^ = 0%, *P* = 0.49). The pooled OR of ε3/ε4 vs. ε3/ε3 was 0.98 (95% CI: 0.73–1.32; *P* = 0.9146), and that of ε4/ε4 vs. ε3/ε3 was 0.83 (95% CI: 0.53–1.28; *P* = 0.3904) (Figs. 4 and 5). For this reason, ε3/ε4 and ε4/ε4 did not show a protective effect on T2DN. However, ε2/ε2 and ε2/ε3 increased T2DN risk (ε2/ε2 vs. ε3/ε3: OR = 2.32, 95% CI: 1.52–3.56, *P* = 0.0001; ε2/ε3 vs. ε3/ε3: OR = 1.97, 95% CI: 1.50–2.59, *P*<0.0001) (Figs. 6 and 7), and ε2/ε4 genotype also increased T2DN risks significantly (ε2/ε4 vs. ε3/ε3: OR = 1.69, 95% CI: 1.18–2.44, *P* = 0.0046) (Fig. 8).

### Subgroup analysis

For *APOE* alleles, when ε2 was compared with ε3, the association of increased T2DN risk was significant in Chinese population (OR = 2.04, 95% CI: 1.58–2.62); however, when ε4 was compared with ε3, the protective association of T2DN risk was significant in other population (OR = 0.68, 95% CI: 0.51–0.91) (Table 2). For *APOE* genotypes, the increased T2DN risks in Chinese population were identified for the genotypes (ε2/ε2 vs. ε3/ε3: OR = 2.74, 95% CI: 1.67–4.49; ε2/ε3 vs. ε3/ε3: OR = 2.09, 95% CI: 1.58–2.76; ε2/ε4 vs. ε3/ε3: OR = 1.64, 95% CI: 1.08–2.50). Whereas, ε3/ε4 genotype decreased T2DN risk in other population (ε3/ε4 vs. ε3/ε3: OR = 0.61, 95% CI: 0.44–0.84), but ε4/ε4 genotype were not associated with T2DN risk in neither of the populations (Table 2). The source of heterogeneity was not found using meta-regression analysis, although each factor decreased overall heterogeneity.

**Table 2:** Subgroup analysis of association between *ApoE* alleles / genotypes and diabetic nephropathy

Variable	China					Other
	*OR*	(95% CI)	*I*^2^ (%)	*OR*	(95%CI)	*I*^2^ (%)
Alleles						
ε2	2.04	(1.58,2.62)	50	1.56	(0.97,2.53)	70
ε4	1.26	(0.94,1.71)	68	0.68	(0.51–0.91)	46
Genotypes						
ε2/ε2	2.74	(1.67, 4.49)	1	1.29	(0.52, 3.16)	6
ε2/ε3	2.09	(1.58, 2.76)	35	1.69	(0.95, 2.99)	69
ε2/ε4	1.64	(1.08, 2.50)	13	1.88	(0.90, 3.91)	33
ε3/ε4	1.46	(0.99, 2.15)	71	0.61	(0.44, 0.84)	38
ε4/ε4	0.80	(0.47, 1.36)	0	0.89	(0.42, 1.89)	6

*ApoE* alleles (ε2 and ε4) and genotypes (ε2/ε2, ε2/ε3, ε2/ε4, ε3/ε4 and ε4/ε4) were compared with ε3 and ε3/ε3

### Sensitivity analysis and publication bias

Results of sensitivity analysis in this meta-analysis revealed that there was no individual article influencing the corresponding pooled ORs and 95% CIs (Table 3 and Table 4), indicating that results of this meta-analysis are robust.

**Table 3:** Sensitivity analysis of association between *ApoE* alleles and diabetic nephropathy

Study	ε2	ε4
Horita et al. [17]	1.84 (1.46, 2.33)	0.96 (0.76, 1.22)
Eto et al. [18]	1.86 (1.47, 2.36)	0.97 (0.76, 1.23)
Kimura et al. [19]	1.94 (1.53, 2.45)	1.00 (0.79, 1.25)
Zhang et al. [20]	1.86 (1.47, 2.37)	0.97 (0.77, 1.23)
Xiang et al. [21]	1.87 (1.47, 2.37)	0.98 (0.77, 1.23)
Ha et al. [22]	1.86 (1.46, 2.35)	0.97 (0.77, 1.22)
Akarsu et al. [23]	1.86 (1.47, 2.35)	0.98 (0.78, 1.23)
Dai et al. [24]	1.92 (1.51, 2.43)	0.96 (0.76, 1.21)
Shen et al. [25]	1.86 (1.47, 2.37)	0.96 (0.76, 1.21)
Zhang et al. [26]	1.84 (1.46, 2.33)	0.97 (0.77, 1.22)
Liu et al. [27]	1.92 (1.51, 2.44)	0.96 (0.76, 1.21)
Park et al. [28]	1.84 (1.46, 2.32)	0.98 (0.78, 1.24)
Liu et al. [29]	1.86 (1.47, 2.35)	0.97 (0.77, 1.22)
Xiong et al. [30]	1.92 (1.52, 2.43)	0.96 (0.76, 1.21)
Hua et al. [31]	1.89 (1.48, 2.40)	0.99 (0.78, 1.25)
Guo et al. [32]	1.85 (1.46, 2.33)	0.97 (0.77, 1.22)
Ng et al. [33]	1.92 (1.50, 2.46)	0.96 (0.76, 1.22)
Zhang et al. [34]	1.86 (1.47, 2.34)	0.95 (0.76, 1.20)
Pan et al. [35]	1.95 (1.54, 2.46)	0.93 (0.75, 1.16)
Ilhan et al. [36]	1.94 (1.55, 2.45)	0.96 (0.76, 1.21)
Kwon et al. [37]	1.91 (1.51, 2.42)	0.99 (0.79, 1.24)
Leiva et al. [38]	1.90 (1.51, 2.40)	1.01 (0.81, 1.26)
Rouzi et al. [39]	1.88 (1.49, 2.39)	0.98 (0.78, 1.23)
Erdogan et al. [40]	1.90 (1.50, 2.40)	0.98 (0.78, 1.23)
Xiang et al. [41]	1.87 (1.48, 2.38)	0.99 (0.78, 1.24)
Reis et al. [42]	1.99 (1.62, 2.45)	0.99 (0.79, 1.25)
Sun et al. [43]	1.92 (1.51, 2.46)	0.93 (0.75, 1.16)
Satirapoj et al. [44]	1.91 (1.50, 2.43)	1.00 (0.80, 1.26)
Wang et al. [45]	1.83 (1.45, 2.31)	0.98 (0.77, 1.23)
Luo et al. [46]	1.82 (1.45, 2.29)	0.91 (0.75, 1.12)
Atta et al. [47]	1.85 (1.46, 2.34)	0.96 (0.76, 1.21)
Jiang et al. [48]	1.87 (1.47, 2.40)	0.98 (0.77, 1.25)
Karimoei et al. [49]	1.91 (1.51, 2.43)	1.00 (0.80, 1.25)

*ApoE* alleles (ε2 and ε4) were compared with ε3

**Table 4:** Sensitivity analysis of association between *ApoE* genotypes and diabetic nephropathy

Study	ε2/ε2	ε2/ε3	ε2/ε4	ε3/ε4	ε4/ε4
Horita et al. [37]	2.27 (1.49, 3.53)	1.91 (1.45, 2.52)	1.70 (1.18, 2.46)	0.99 (0.73, 1.34)	0.73 (0.46, 1.16)
Eto et al. [36]	2.27 (1.50, 3.55)	1.96 (1.48, 2.59)	1.68 (1.16, 2.43)	0.99 (0.72, 1.34)	0.79 (0.50, 1.25)
Kimura et al. [39]	2.27 (1.52, 3.56)	2.01 (1.52, 2.66)	1.86 (1.28, 2.71)	1.01 (0.75, 1.36)	0.84 (0.54, 1.31)
Zhang et al. [30]	2.27 (1.41, 3.36)	1.99 (1.50, 2.63)	1.67 (1.15, 2.42)	0.99 (0.73, 1.34)	0.83 (0.53, 1.28)
Xiang et al. [45]	2.27 (1.48, 3.52)	1.96 (1.48, 2.59)	1.72 (1.19, 2.48)	0.99 (0.73, 1.34)	0.82 (0.53, 1.28)
Ha et al. [46]	2.27 (1.46, 3.47)	1.94 (1.47, 2.57)	1.73 (1.19, 2.50)	0.97 (0.72, 1.32)	0.83 (0.54, 1.30)
Akarsu et al. [47]	2.27 (1.48, 3.50)	1.96 (1.49, 2.59)	1.69 (1.18, 2.44)	0.99 (0.74, 1.34)	0.82 (0.53, 1.27)
Dai et al. [24]	2.27 (1.52, 3.56)	2.02 (1.53, 2.67)	1.70 (1.18, 2.46)	0.97 (0.72, 1.31)	0.83 (0.53, 1.29)
Shen et al. [32]	2.27 (1.54, 3.66)	1.91 (1.45, 2.51)	1.73 (1.19, 2.51)	0.95 (0.71, 1.28)	0.84 (0.54, 1.31)
Zhang et al. [33]	2.27 (1.47, 3.49)	1.94 (1.47, 2.56)	1.66 (1.15, 2.40)	1.00 (0.74, 1.35)	0.80 (0.51, 1.24)
Liu et al. [42]	2.27 (1.53, 3.61)	2.02 (1.52, 2.68)	1.72 (1.19, 2.49)	0.97 (0.72, 1.32)	0.82 (0.53, 1.27)
Park et al. [23]	2.27 (1.52, 3.56)	1.91 (1.45, 2.50)	1.69 (1.18, 2.44)	1.00 (0.74, 1.35)	0.83 (0.53, 1.28)
Liu et al. [34]	2.27 (1.52, 3.56)	1.94 (1.47, 2.56)	1.69 (1.17, 2.44)	0.98 (0.73, 1.32)	0.83 (0.53, 1.28)
Xiong et al. [29]	2.27 (1.54, 3.65)	2.02 (1.53, 2.66)	1.71 (1.18, 2.47)	0.98 (0.73, 1.32)	0.80 (0.51, 1.25)
Hua et al. [27]	2.27 (1.56, 3.74)	1.91 (1.45, 2.52)	1.86 (1.27, 2.71)	1.00 (0.74, 1.35)	0.82 (0.52, 1.28)
Guo et al. [31]	2.27 (1.41, 3.36)	1.95 (1.48, 2.56)	1.74 (1.19, 2.54)	0.98 (0.73, 1.32)	0.83 (0.53, 1.28)
Ng et al. [43]	2.27 (1.58, 3.90)	2.02 (1.50, 2.71)	1.78 (1.21, 2.60)	0.97 (0.71, 1.31)	0.85 (0.55, 1.33)
Zhang et al. [25]	2.27 (1.47, 3.48)	1.97 (1.49, 2.60)	1.67 (1.16, 2.42)	0.95 (0.71, 1.28)	0.84 (0.54, 1.30)
Pan et al. [26]	2.27 (1.58, 3.78)	2.03 (1.54, 2.69)	1.71 (1.18, 2.47)	0.93 (0.70, 1.23)	0.80 (0.51, 1.24)
Ilhan et al. [38]	2.27 (1.64, 3.98)	2.01 (1.52, 2.65)	1.69 (1.18, 2.44)	0.97 (0.72, 1.31)	0.83 (0.53, 1.28)
Kwon et al. [40]	2.27 (1.52, 3.56)	2.03 (1.53, 2.67)	1.68 (1.16, 2.43)	1.02 (0.76, 1.36)	0.84 (0.54, 1.30)
Leiva et al. [41]	2.27 (1.52, 3.56)	2.00 (1.52, 2.63)	1.69 (1.18, 2.44)	1.03 (0.77, 1.38)	0.86 (0.55, 1.34)
Rouzi et al. [15]	2.27 (1.47, 3.49)	1.96 (1.49, 2.58)	1.82 (1.24, 2.65)	0.98 (0.73, 1.32)	0.83 (0.53, 1.28)
Erdogan et al. [35]	2.27 (1.52, 3.56)	2.01 (1.53, 2.65)	1.66 (1.14, 2.39)	1.01 (0.75, 1.36)	0.83 (0.53, 1.28)
Xiang et al. [28]	2.27 (1.52, 3.56)	1.95 (1.47, 2.58)	1.74 (1.20, 2.51)	1.00 (0.74, 1.35)	0.83 (0.53, 1.28)
Reis et al. [44]	2.27 (1.52, 3.56)	2.09 (1.66, 2.64)	1.69 (1.18, 2.44)	1.02 (0.75, 1.37)	0.83 (0.53, 1.28)
Sun et al. [16]	2.55 (1.60, 4.05)	2.03 (1.52, 2.71)	1.55 (1.07, 2.25)	0.94 (0.70, 1.27)	0.83 (0.53, 1.28)
Satirapoj et al. [22]	2.27 (1.59, 3.82)	2.00 (1.51, 2.66)	1.69 (1.18, 2.44)	1.02 (0.76, 1.37)	0.83 (0.53, 1.28)
Wang et al. [17]	2.27 (1.39, 3.32)	1.95 (1.48, 2.58)	1.66 (1.14, 2.41)	1.01 (0.75, 1.36)	0.82 (0.52, 1.30)
Luo et al. [18]	2.27 (1.38, 3.29)	1.89 (1.45, 2.46)	1.50 (1.03, 2.18)	0.92 (0.71, 1.18)	0.75 (0.48, 1.18)
Atta et al. [19]	2.27 (1.52, 3.56)	1.92 (1.47, 2.50)	1.47 (1.01, 2.15)	0.98 (0.73, 1.32)	0.83 (0.53, 1.28)
Jiang et al. [20]	2.27 (1.43, 3.48)	1.98 (1.48, 2.65)	1.66 (1.13, 2.44)	0.99 (0.72, 1.36)	1.03 (0.63, 1.66)
Karimoei et al. [21]	2.27 (1.58, 3.78)	1.99 (1.50, 2.63)	1.76 (1.21, 2.55)	1.02 (0.75, 1.37)	0.87 (0.56, 1.36)

*ApoE* genotypes (ε2/ε2, ε2/ε3, ε2/ε4, ε3/ε4 and ε4/ε4) were compared with ε3/ε3

Beggʼs funnel plot and Eggerʼs test identified that significant publication bias was not found between either allele and either genotype and T2DN risk (all *P*>0.05). (Supplementary Figure S1).

### Trial sequential analysis

With regard to the relationship of ε2 with T2DN risks and for the relationship of the genotypes (ε2/ε2, ε2/ε3, and ε2/ε4) with T2DN risks, the sample size reached RIS, and the Z-curve crossed the trial sequential monitoring boundary (Supplementary Figure S2). For the relationship of the ε4/ε4 genotype with T2DN risks, the sample size reached RIS (Supplementary Figure S3). For the relationship of ε4 with T2DN risks and for the relationship of the ε3/ε4 genotype with T2DN risks, the sample size and Z curve were not up to the requirements (Supplementary Figure S3).

## Discussion

This meta-analysis further investigated the association between the *APOE* polymorphism and T2DN risks using up-to-date data, indicating that ε2 allele may increase T2DN risks; moreover, ε2/ε2, ε2/ε3, and ε2/ε4 genotypes increase T2DN risks. The ε2 allele and the ε2-involved genotypes may confer the association of *APOE* polymorphism with T2DN risk.

Meta-analyses between ε2/ε3/ε4 of *APOE* and DN risks have been performed to recognize the function of variants in *APOE*. In 2011, Li et al. found that ε2 increases T2DN risk in patients with diabetes [50]. In 2014, Lin et al. also showed that ε2 polymorphism increased the susceptibility to T2DN in Asian population [10]. In 2015, Li et al. validated that ε2 may act as promotion factors of nephropathy in type 2 diabetes, but ε4 is not associated with T2DN risk [12]. This meta-analysis further corroborated that the ε2 allele and the ε2-involved genotypes may confer the association of *APOE* genetic polymorphism with T2DN risk. Additionally, the association of ε2 with increased T2DN risks was further identified in Chinese population, and ε4 and ε3/ε4 genotype were associated with decreased T2DN risks in other population.

Heterogeneity affects interpretations of results [51]. Although the source was not pinpointed, each separate factor did decrease the overall heterogeneity. Sensitivity analyses and TSA were further performed to assess the robustness of the deductions, reflecting a reliable conclusion.

Oxidative stress affects APOE via amino acid residues 112 and 158, suggesting that oxidative stress may be a source of heterogeneity [52]. Reduced glutathione provides major antioxidative activity; however, glutathione levels were remarkably reduced in patients with DN compared with those in patients with diabetes and healthy controls [53]. The meta-analysis documented the relationship of ε2 allele and the genotypes (ε2/ε2, ε2/ε3, and ε2/ε4) with T2DN risk, suggesting that APOE2 in patients with T2DN cannot balance oxidative stress involved in T2DN progress, and oxidative stress may generate heterogeneity in patients with T2DN.

APOE is interfered by oxidative stress in structure and function. APOE contains two domains (the low-density-lipoprotein receptor [LDLR] binding region [residues 136–150] and the principal lipoprotein-binding region [residues 244–272]), highlighting the implication of the LDLR-binding region of APOE in DN progress. The affinity of APOE3 to LDLR is similar to that of APOE4; however, the binding ability of APOE2 is significantly lower [54]. Moreover, the cysteine-to-arginine substitution in APOE2 at position 158 affects LDLR-binding activity by forming of a new salt bridge between Arg150 and Asp154, further affecting the interaction between APOE2 and LDLR [55]. Thus, oxidative stress interferes the structure and function of APOE by dysregulating the affinity of APOE to LDLR possibly, and the dysregulation of LDLR correlates with DN risk directly [56]. Furthermore, renal lipid accumulation is observed in human DN [57], and knockout of *ApoE* increases foam cell-rich soft plaques and aggressive renal dysfunction in mice substantially [58].

### Study strengths and limitations

There are some strengths in this study. First, the up-to-date articles were collected extensively, rendering this study more statistical power to draw valid conclusion on this issue. Second, TSA was the first utilized to evaluate the association of *APOE* genetic polymorphism with T2DN risk, conferring reliable evidence to reach the conclusion.

Some limitations exist in this study. First, the main source of heterogeneity was not identified, although subgroup analysis and regression analysis were conducted, and further studies based on larger sample size and multiple ethnicity and region are required. Moreover, the other factors, which could contribute to heterogeneity, are not retrieved. Second, data of oxidative stress status, which possibly reflects renal injury more directly than *APOE* genetic polymorphism, are not available in literatures. Third, the case-control design could prove an association, rather than a causal relationship, thereby needing prospective cohort studies in future.

## Conclusion

In conclusion, the ε2 allele and the ε2-involved genotypes (ε2/ε2, ε2/ε3, and ε2/ε4) may confer the association of *APOE* genetic polymorphism with T2DN risk. Investigations of oxidative stress status in blood of patients with T2DN are necessary for giving more insight into the association. Elucidating the risk factors of T2DN would be meaningful for the mechanism and control of the disease.

## Supplementary information

**Additional file 1: Figure S1.** Funnel plot of the association between *ApoE* gene polymorphism and nephropathy in type 2 diabetes. (A) *ε*2 allele (B) *ε*4 allele (C) *ε*2/*ε*2 genotype (D) *ε*2/*ε*3 genotype (E) *ε*2/*ε*4 genotype (F) *ε*3/*ε*4 genotype (G)*ε*4/*ε*4 genotype. **Figure S2.** Trial sequential analysis of the association between *ApoE* gene polymorphism and nephropathy in type 2 diabetes. (A) *ε*2 allele; (B) ε2/ε2 genotype; (C) *ε*2/*ε*3 genotype; (D) *ε*2/*ε*4 genotype. **Figure S3.** Trial sequential analysis of the association between *ApoE* gene polymorphism and nephropathy in type 2 diabetes. (A) *ε*4 allele; (B) *ε*3/*ε*4 genotype; (C) *ε*4/*ε*4 genotype.

## Data Availability

All data generated or analysed during this study are included in this published article.

## Abbreviations

DM: Diabetes mellitus
DN: Diabetic nephropathy
T2DN: Nephropathy in type 2 diabetes
ROS: Reactive oxygen species
APOE: Apolipoprotein E
SNPs: Single nucleotide polymorphisms
NOS: Newcastle-Ottawa scale
HWE: Hardy–Weinberg equilibrium
OR: Odds ratios
95% CI: 95% confidence intervals
TSA: Trial sequential analysis
RIS: Required information size
LDLR: Low-density-lipoprotein receptor.
