# Supplementary material for: ε2 allele and ε2-involved genotypes (ε2/ε2, ε2/ε3, and ε2/ε4) may confer the association of APOE genetic polymorphism with risks of nephropathy in type 2 diabetes: a meta-analysis
Source: Lipids Health Dis. 2020 Jun 13;19:136. doi: 10.1186/s12944-020-01307-6 (PMC7293775; doi:10.1186/s12944-020-01307-6)
Supplement: Supplementary file 1 — Additional file 1: Figure S1. Funnel plot of the association between ApoE gene polymorphism and nephropathy in type 2 diabetes. (A) ε2 allele (B) ε4 allele (C) ε2/ε2 genotype (D) ε2/ε3 genotype (E) ε2/ε4 genotype (F) ε3/ε4 genotype (G)ε4/ε4 genotype. Figure S2. Trial sequential analysis of the association between ApoE gene polymorphism and nephropathy in type 2 diabetes. (A) ε2 allele; (B) ε2/ε2 genotype; (C) ε2/ε3 genotype; (D) ε2/ε4 genotype. Figure S3. Trial sequential analysis of the association between ApoE gene polymorphism and nephropathy in type 2 diabetes. (A) ε4 allele; (B) ε3/ε4 genotype; (C) ε4/ε4 genotype. [file 12944_2020_1307_MOESM1_ESM.docx]

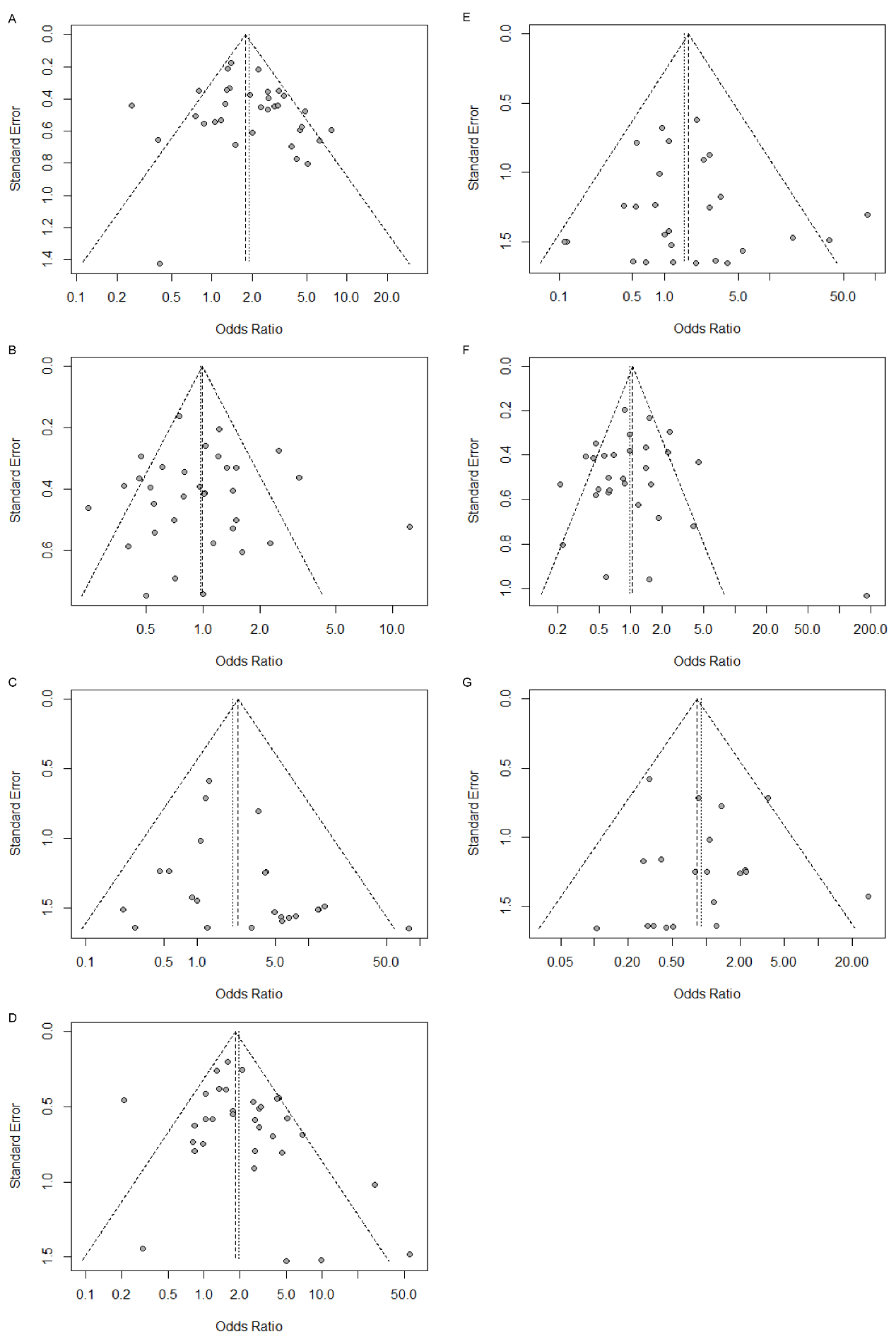


**Supplementary Figure S1**. Funnel plot of the association between *ApoE* gene polymorphism and nephropathy in type 2 diabetes. (A) *ε*2 allele (B) *ε*4 allele (C) *ε*2/*ε*2 genotype (D) *ε*2/*ε*3 genotype (E) *ε*2/*ε*4 genotype (F) *ε*3/*ε*4 genotype (G)*ε*4/*ε*4 genotype.


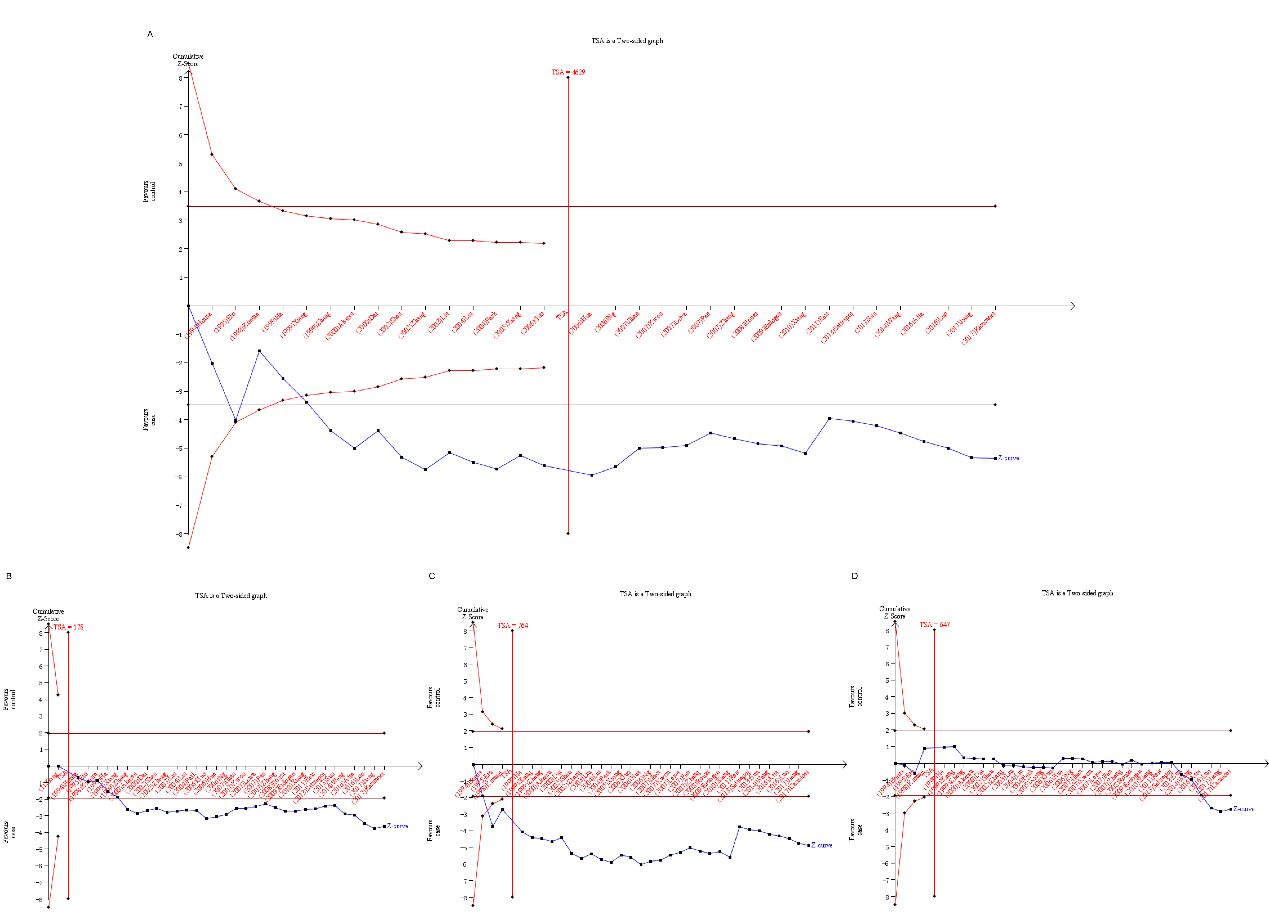


**Supplementary Figure S2**. Trial sequential analysis of the association between *ApoE* gene polymorphism and nephropathy in type 2 diabetes. (A) *ε*2 allele; (B) ε2/ε2 genotype; (C) *ε*2/*ε*3 genotype; (D) *ε*2/*ε*4 genotype.


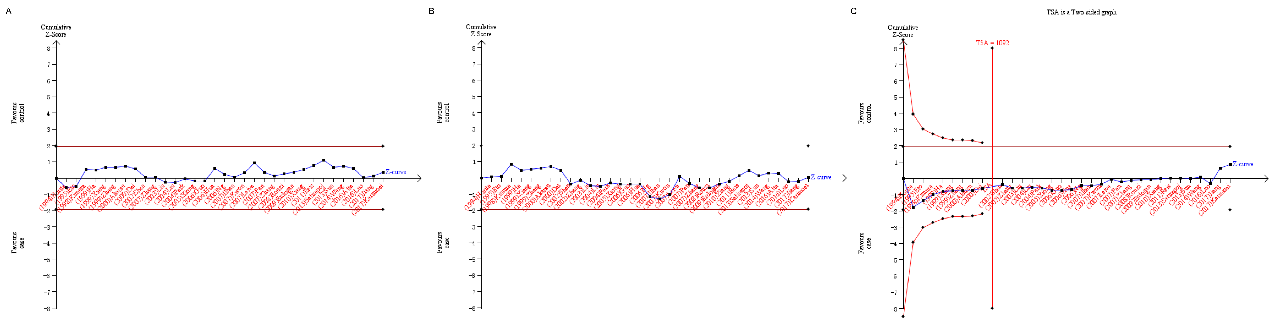


**Supplementary Figure S3**. Trial sequential analysis of the association between *ApoE* gene polymorphism and nephropathy in type 2 diabetes. (A) *ε*4 allele; (B) *ε*3/*ε*4 genotype; (C) *ε*4/*ε*4 genotype.
